# Therapeutic Effects of *Cephalotaxus harringtonia* Leaf Extract on Hepatocellular Carcinoma via Regulation of the Intrinsic Apoptosis Pathway and Cell Cycle

**DOI:** 10.3390/cimb47120994

**Published:** 2025-11-27

**Authors:** Dae-Han Park, Sonny C. Ramos, Hyun Bo Sim, Ju-Bin Lee, Ho-Yeol Jang, Beom-Gyun Jeong, Kyung-Wuk Park, Kyung-Yun Kang, Jong-Jin Kim

**Affiliations:** 1 Department of Biomedical Science, Sunchon National University, 255, Jungang-ro, Suncheon-si 57922, Republic of Korea; carbdsinc@naver.com (D.-H.P.); ynnosomarc@gmail.com (S.C.R.); kokonun3@naver.com (H.B.S.); dlwnqls0526@naver.com (J.-B.L.); 2 R&D Team, Suncheon Research Center for Bio Health Care, Suncheon-si 57962, Republic of Korea; yeol2686@naver.com (H.-Y.J.); fusionchef@sbrc.kr (B.-G.J.); uk988446@sbrc.kr (K.-W.P.); 3 Future of Natural New Materarials, Room 605, Industry-Academic Cooperation Building, 255, Jungang-ro, Suncheon-si 57922, Republic of Korea

**Keywords:** *Cephalotaxus harringtonia*, apoptosis, intrinsic pathway, cell cycle

## Abstract

Apoptosis induction in tumor cells is a fundamental therapeutic approach in cancer treatment, with growing interest in plant-derived compounds that offer potent efficacy and reduced toxicity. *Cephalotaxus harringtonia*, traditionally used in East Asian medicine, contains several bioactive constituents, including homoharringtonine (HHT) and quercetin 3-β-D-glucoside (Q3G), which are known for their anticancer properties. This study investigated the anticancer effects of *C. harringtonia* leaf extract (CHLE) and its two major compounds, quercetin 3-β-D-glucoside (Q3G) and HHT, against human liver cancer cell lines (HepG2). CHLE exhibited selective cytotoxicity and apoptosis specifically in HepG2 cells while showing minimal toxicity toward normal kidney cells (HK-2). Mechanistic analyses revealed that CHLE induced apoptosis through a mitochondria-mediated intrinsic pathway, characterized by increased reactive oxygen species production, mitochondrial membrane depolarization, and BAX upregulation. These findings demonstrate that *C. harringtonia* leaf extract possesses potent, selective anticancer activity and may serve as a promising natural candidate for the prevention and therapeutic management of liver cancer.

## 1. Introduction

Cancer is a global disease characterized by uncontrolled proliferation, migration, and invasion, often resulting from mutations in cell cycle regulatory proteins (cyclin-dependent kinases and cyclins) and oncogenes (*MYC* and *p53*) [1,2,3]. Despite advances in medical technology, the number of deaths caused by lung cancer, colon cancer, stomach cancer, and liver cancer remains high [3,4]. Modern anticancer drugs have developed various strategies, including inducing apoptosis, inhibiting migration, and inhibiting cell proliferation [5,6]. Specifically, apoptosis induction is an evaluated promising approach for removing cancer cells [7]. Consequently, interest in plant-derived extracts known for their fewer side effects has been recently increasing [8].

A member of the Cephalotaxaceae family, *Cephalotaxus harringtonia* (Knight ex J.Forbes) K. Koch (*C. harringtonia*) [9,10], is a small, evergreen coniferous tree or shrub in the mountainous regions of the Republic of Korea, Japan, China, and Taiwan [11,12]. *C. harringtonia* contains various bioactive compounds, including alkaloids, flavonoids, terpenoids, and lignans, and is frequently used in traditional medicine to treat rheumatism and malaria [13,14,15]. These compounds exhibit neuroprotective, anti-inflammatory, antioxidant, and anticancer properties [16,17]. Traditional applications of the extract conceptually overlap with modern pharmacological observations of antitumor and anti-inflammatory activity. Various studies also note that alkaloids from *Cephalotaxus* are major contributors to the reported ethnomedicinal efficacy [15,18]. This ethnopharmacological background suggests that *C. harringtonia* extract may harbor both direct cytotoxic activity and modulatory effects on oxidative stress or cell signaling, providing a meaningful bridge between traditional practice and mechanistic cancer pharmacology.

Homoharringtonine (HHT), belonging to the alkaloid family, inhibits protein translation and has been approved by the Food and Drug Administration (FDA) for the treatment of chronic myeloid leukemia (CML) [19]. This specifically binds to the A site of eukaryotic 60S ribosomes, preventing the access of aminoacyl-tRNA and thereby inhibiting the production of new proteins essential for cell proliferation and survival [20]. Notably, it preferentially affects short-lived regulatory proteins such as Mcl-1, cyclin D1, and c-Myc [21]. In hematologic malignancies, HHT has been shown to downregulate anti-apoptotic Mcl-1, activate caspases, and trigger mitochondrial outer membrane permeabilization [22]. In solid tumor models, HHT has also been reported to modulate several signaling pathways. For example, in non-small-cell lung cancer, it inhibited IL-6/JAK/STAT3 signaling, leading to apoptosis via the mitochondrial pathway [23]. In hepatocellular carcinoma, HHT has been linked to activation of Hippo pathway signaling to suppress proliferation and induce apoptosis [24,25]. Similarly, in colon cancer models, HHT induced S-phase arrest and apoptosis [26].

Q3G (isoquercetin) is a flavonoid with a glucose moiety attached at the 3-O position. Found in various fruits, vegetables, and plants, this compound has demonstrated antioxidant and anti-inflammatory effects [27,28]. In 2021, it was reported to induce apoptosis through S-phase arrest and activation of caspase 3/9 in human ovarian cervical cancer cells (HeLa) [29].

According to previous reports, *C. harringtonia* leaf extract (CHLE) contains higher concentrations of HHT than the *C. harringtonia* stem extracts at 246% [12]. However, the anticancer effects of CHLE on adherent cells have not yet been studied. Therefore, in this study, we evaluated its anticancer effects on adherent liver cell lines (HepG2). HPLC analysis revealed that quercetin 3-β-D-glucoside (Q3G), abundant in CHLE, along with HHT, a compound with reported anticancer effects, were the main substances. Therefore, CHLE, Q3G, and HHT were used to measure cytotoxicity and intrinsic pathway activation.

## 2. Materials and Methods

### 2.1. Materials

Q3G and HHT were purchased from Merck (Darmstadt, Germany) and Sigma-Aldrich (Stockholm, Sweden), respectively, and all samples were dissolved in Dimethyl Sulfoxide (DMSO; Sigma-Aldrich, Stockholm, Sweden).

The following reagents were used for cell culture: RPMI 1640 (Hyclone, Novato, CA, USA); Dulbecco’s Modified Eagle Medium (DMEM)/High glucose (Hyclone, Novato, CA, USA); DMEM/F12 (Hyclone, Novato, CA, USA); fetal bovine serum (FBS; Hyclone, Novato, CA, USA); 2-mercaptoethanol (2-ME; Thermo, Waltham, MA, USA); and antibiotic-antimycotic (Thermo, Waltham, MA, USA).

The following reagents were used for cytotoxicity and cellular response analyses: FxCycle™ PI/RNase Staining Solution (Thermo, Waltham, MA, USA); Cell Counting Kit-8 (CCK-8; Dojindo Lab., Kumamoto, Japan); RNeasy Mini kit (Qiagen, Valencia, CA, USA); SYBR^®^ Green Realtime Master Mix kit (Thermo, Waltham, MA, USA); FITC Annexin V Apoptosis Detection Kit I (BD Biosciences, San Jose, CA, USA); 2′,7′-dichlorodihydrofluorescein diacetate (DCFDA)/H2DCFDA Cellular ROS Assay Kit (Abcam, Cambridge, UK); MitoProbe JC-1 (5, 5′, 6, 6′-tetrachloro-1, 1′, 3, 3′-tetraethylbenzimidazol-carbocyanine iodide) Assay kit (Molecular Probes, Eugene, OR, USA); FoxP3 Staining Buffer Kit (Miltenyi Biotec, Auburn, CA, USA); FITC-conjugated anti-BAX antibody (clone B-9; Santa Cruz Biotechnology, Santa Cruz, CA, USA); and PE-conjugated anti-BCL-2 antibody (clone SC-509; Santa Cruz Biotechnology, Santa Cruz, CA, USA).

The following cell lines were used in this study: human liver cancer cell line (HepG2; KCLB 88065); human lung cancer cell line (A549; KCLB 10185); human colon cancer cell line (HCT116; KCLB 10247); human stomach cancer cell line (AGS; KCLB 21739); and the human normal kidney cell line (HK-2; KCLB 22190). All cell lines were purchased from the Korean Cell Line Bank (KCLB, Seoul, Republic of Korea).

### 2.2. Plant Materials, Pretreatment, and Extraction of Samples

The leaves of *C. harringtonia* samples were collected from Baegunsan Mountain, located at San 25, Jukcheon-ri, Okryong-myeon, Gwangyang-si, Jeollanam-do, Republic of Korea (Latitude: 35°03′23.3″ N; Longitude: 127°37′53.3″ E), from May to July 2022. The collected plant material was taxonomically confirmed as *C. harringtonia* by the miDNA Genome Research Institute (Gunsan-si, Jeonbuk Province, Republic of Korea), and molecular identification was performed using ITS sequencing (Thermo, Waltham, MA, USA), with the corresponding sequence deposited in GenBank under accession number MK116531.1. A voucher specimen was authenticated and archived at the miDNA Genome Research Institute herbarium for future reference.

*C. harringtonia* leaves were thoroughly washed with purified water and air-dried at room temperature for 48 h. The extraction process was carried out by adding the washed and dried leaves to purified water (10 g/800 mL) and extracting them at 85 °C for 3 h using a hot water reflux extraction device. The extract was cooled to room temperature and filtered through filter paper (Wattman paper filter No. 2) when it reached 25 °C, and then filtered through a polypropylene membrane. The filtrate was freeze-dried to obtain the *C. harringtonia* leaf extract with a final yield of 14.78%. The freeze-dried extract used in in vitro experiments was stored at −20 ± 2 °C before use [30].

### 2.3. Quantification of Phytochemicals (Condensed Tannins, Flavonoids, and Phenolics)

Phytochemical compounds were quantified using established colorimetric methods: condensed tannins by modified vanillin assay (expressed as μg catechin equivalents/mg dry weight), total flavonoids by aluminum chloride method with quercetin standard (1–500 μg/mL range, expressed as μg QE/mg dry weight), and total phenolics by modified Folin–Denis method with gallic acid standard (1–500 μg/mL range, expressed as μg GAE/mg dry weight). All protocols were optimized for sample characteristics following established procedures [31,32,33].

### 2.4. HPLC-ELSD/MS Analysis

Standard preparation: Stock solutions of HHT and Q3G (1 mg/mL) were prepared in methanol. Aliquots (200 μL) were transferred to autosampler vials and maintained at 24 °C for throughout analysis.

Instrumentation: Analysis was performed using an Agilent 1260 HPLC (Agilent, Santa Clara, CA, USA). system coupled with 380 ELSD and 6130 quadrupole mass spectrometer. Chromatographic separation was achieved on a Poroshell 120 SB-C18 column (150 × 4.6 mm, 2.7 μm) with a C18 guard column at 24 °C and a 0.4 mL/min flow rate.

Chromatographic conditions: Mobile phase consisted of water (A) and acetonitrile (B), both containing 0.01% trifluoroacetic acid. Gradient elution: 0–3 min (95% A), 3–20 min (linear to 100% B), 20–30 min (100% B). Injection volume: 10 μL.

MS parameters: Electrospray ionization operated in positive/negative modes with optimized conditions: capillary voltage 3000 V, drying gas 12.0 L/min at 350 °C, nebulizer pressure 35 psig, fragmentor voltage 70 V, mass range 100–1000 m/z in scan mode. ELSD conditions: gas flow 1.60 SLM, LED intensity 100%, evaporator and nebulizer temperatures 30 °C. UV Parameters: HHT 290nm and Q3G 254 nm [34].

### 2.5. Cell Culture

The A549 and HCT116 cells were cultured in RPMI 1640, the HepG2 and AGS cells were cultured in DMEM/High glucose, and the HK-2 cells were cultured in DMEM/F12. Each medium was supplemented with 10% FBS, 0.1% 2-ME, 25 mM HEPES, 1% sodium pyruvate, and 1% antibiotic-antimycotic solution. All cells were maintained at 37 °C under 5% CO_2_ incubator.

### 2.6. C. harringtonia Leaf Extract Solution Preparation

Freeze-dried CHLE was dissolved in DMSO at room temperature for 8 h, and only the supernatant was collected and used. The final stock concentration of CHLE was stored at 10 or 100 mg/mL. The DMSO content of the highest CHLE concentration (10 or 100 µg/mL) used in the experiment was 0.1%.

### 2.7. Cell Cycle Assay

HepG2, A549, HCT116, and AGS cells were seeded in 24-well plates (5 × 10^4^ cells/well), incubated for 24 h, and treated with CHLE (1, 3, and 10 µg/mL) and positive control DMSO (0.1%) for 24 h or 48 h. After treatment, the cells were harvested, fixed in 70% ethanol for 30 min, and stained with FxCycle™ PI/RNase staining solution for 10 min. The cell cycle was analyzed by flow cytometry (FACS Canto II, BD Biosciences, San Jose, CA, USA). Data analysis was performed using FlowJo software (version 10.10.0; TreeStar, Woodburn, OR, USA) [35].

### 2.8. Cytotoxic Assay

HepG2 and HK-2 cells were seeded in 96-well plates (5 × 10^3^ cells/well), incubated for 24 h, and treated with CHLE (1, 3, 10, 30, and 100 µg/mL), Q3G (3 and 10 µg/mL), and HHT (0.01 and 0.03 µg/mL) for 24 h or 48 h. CCK-8 was used to analyze cell cytotoxicity. Absorbance was measured at 450 nm using a microplate reader (VersaMax, Molecular Devices, San Jose, CA, USA) [35].

### 2.9. Cell Image

HepG2 cells were seeded in 24-well plates (5 × 10^4^ cells/well), incubated for 24 h, and treated with CHLE (10 µg/mL), Q3G (10 µg/mL), HHT (0.03 µg/mL), and positive control DMSO (0.1%) for up to 48 h. Images were captured at 0, 24, and 48 h using the EVOS M7000 imaging system (10× air objective; Thermo, Waltham, MA, USA). The images were analyzed using Celleste image analysis software (version 6.0; Thermo, Waltham, MA, USA) [36].

### 2.10. Quantitative Reverse Transcription Polymerase Chain Reaction (qRT-PCR)

HepG2 cells were seeded in 6-well plates (5 × 10^5^ cells/well), incubated for 24 h, and treated with CHLE (10 µg/mL) and positive control DMSO (0.1%) for 24 h. The total RNA from cell lines was purified using a RNeasy Mini kit (Qiagen, Valencia, CA, USA). RNA was eluted in ribonuclease (RNase)-free water, quantified using a NanoDrop 2000 (Thermo, Wilmington, DE, USA), and diluted to 100 ng/3 µL in RNase-free water. qRT-PCR using RNA-direct SYBR^®^ Green Realtime Master Mix kit (Thermo, Waltham, MA, USA) was performed according to the manufacturer’s instructions with modifications [36].

The qRT-PCR primers are as follows:

GAPDH Forward: GTCTCCTCTGACTTCAACAGCG
GAPDH Reverse: ACCACCCTGTTGCTGTAGCCAA
Cyclin E Forward: TGTGTCCTGGATGTTGACTGCC
Cyclin E Reverse: CTCTATG TCGCACCACTGATACC
CDK2 Forward: ATGGATGCCTCTGCTCTCACTG
CDK2 Reverse: CCCGATGAGAATGGCAGAAAGC

### 2.11. Annexin V/Propidium Iodide (PI) Staining Assay

HepG2 cells were seeded in 24-well plates (5 × 10^4^ cells/well), incubated for 24 h, and treated with CHLE (1, 3, and 10 µg/mL), Q3G (3 and 10 µg/mL), HHT (0.01 and 0.03 µg/mL), and positive control DMSO (0.1%) for 24 h or 48 h. Apoptotic cells were quantified by flow cytometry (FACS Canto II, BD Biosciences, San Jose, CA, USA) using the FITC Annexin V Apoptosis Detection Kit I (BD Biosciences, San Jose, CA, USA) according to the manufacturer’s instructions. Data were analyzed using FlowJo software (version 10.10.0; TreeStar, Woodburn, OR, USA) [37].

### 2.12. Measurement of Reactive Oxygen Species (ROS) Formation

ROS levels were detected using the DCFDA/H2DCFDA-Cellular ROS Assay Kit (Abcam, Cambridge, UK), according to the manufacturer’s protocol. HepG2 cells were seeded in 24-well plates (5 × 10^4^ cells/well), incubated for 24 h, and treated with CHLE (1, 3, and 10 µg/mL), Q3G (3 and 10 µg/mL), HHT (0.01 and 0.03 µg/mL), and positive control DMSO (0.1%) for 24 h or 48 h. The mean fluorescence intensity (MFI) of DCFDA was measured by flow cytometry (SA3800 Cell Analyzer, Sony Corp., Tokyo, Japan). Data were analyzed using FlowJo software (version 10.10.0; TreeStar, Woodburn, OR, USA) [36].

### 2.13. Mitochondrial Membrane Potential (MMP) Assay

MMP was measured using the MitoProbe JC-1 assay kit (Molecular Probes, Eugene, OR, USA) according to the manufacturer’s protocol. HepG2 cells were seeded in 24-well plates (5 × 10^4^ cells/well), incubated for 24 h, and treated with CHLE (1, 3, and 10 µg/mL), Q3G (3 and 10 µg/mL), HHT (0.01 and 0.03 µg/mL), and positive control DMSO (0.1%) for 24h or 48 h. The cells with lower MMP were measured by flow cytometry (FACS Canto II, BD Biosciences, San Jose, CA, USA) using PE (Ex 565/Em 578) and FITC (Ex 490/Em 525) wavelengths. Data were analyzed using FlowJo software (version 10.10.0; TreeStar, Woodburn, OR, USA) [37].

### 2.14. Measurement of BAX and BCL-2 Expression

HepG2 cells were seeded in 24-well plates (5 × 10^4^ cells/well), incubated for 24 h, and treated with CHLE (1, 3, and 10 µg/mL), Q3G (3 and 10 µg/mL), HHT (0.01 and 0.03 µg/mL), and positive control DMSO (0.1%) for 24 h or 48 h. After fixation and permeabilization using the FoxP3 Staining Buffer Kit (Miltenyi Biotec, Auburn, CA, USA), the cells were stained with FITC-conjugated anti-BAX antibody and PE-conjugated anti-BCL-2 antibody. The expression levels of BAX and BCL-2 were measured by flow cytometry (FACS Canto II, BD Biosciences, San Jose, CA, USA). Data were analyzed using FlowJo software (version 10.10.0; TreeStar, Woodburn, OR, USA) [38].

### 2.15. Statistical Analysis

Statistical analysis was performed using one-way analysis of variance (ANOVA) followed by Tukey’s post hoc test. Statistical calculations were conducted using SPSS software (version 27; SPSS, Chicago, IL, USA). A *p* < 0.05 was considered statistically significant. All data are presented as the mean ± standard deviation (SD).

## 3. Results

### 3.1. Quantification of Condensed Tannins, Flavonoids, and Phenolics

The concentrations of total phenolics, flavonoids, and condensed tannins in CHLE were quantified using standard calibration curves prepared with gallic acid, quercetin, and catechin, respectively. Specifically, CHLE contained 48.93 ± 0.97 μg GAE/mg of total phenolics, 306.28 ± 5.02 μg QE/mg of flavonoids, and 35.73 ± 1.96 μg CE/mg of condensed tannins.

### 3.2. HPLC–ELSD/MS Analysis of CHLE

**Figure 1:**
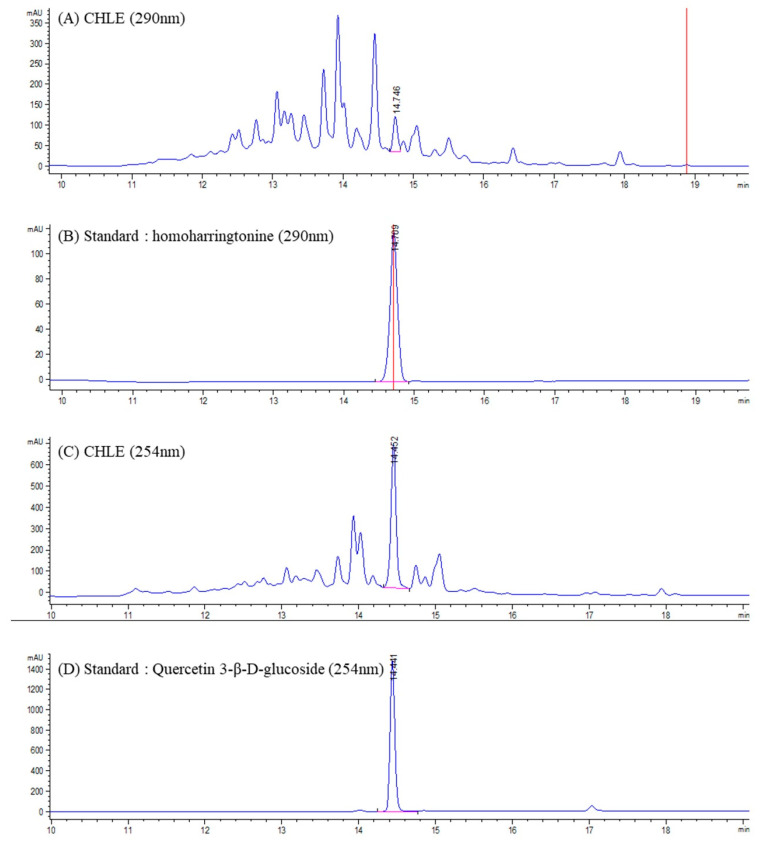
Chromatograms of a sample (CHLE) and standard: (**A**) *C. harringtonia* leaf extract (CHLE_290 nm); (**B**) Homoharringtonine (290 nm) standard: (**C**) *C. harringtonia* leaf extract (CHLE_254 nm); (**D**) Quercetin 3-β-D-glucoside (254 nm).

CHLE and the major compounds were analyzed using LC–ELSD/MS. The mass spectrometric conditions were optimized to enhance the sensitivity toward compounds with similar structural features, thereby allowing reliable detection of the target peak. Accordingly, positive ion mode was employed, in which most constituents exhibited quasi-molecular ions of (M+H)^+^ and (M+Na)^+^. The combined LC–MS approach provided simultaneous UV and MS information for each chromatographic peak. In most cases, direct identification was achieved by comparing retention times, UV spectra, and *m*/*z* values with either published data or authentic standards. Based on these comparisons, CHLE was unambiguously identified as HHT (retention time: 14.746 min) and Q3G (retention time: 14.452 min) (Figure 1). In positive ion mode, HHT exhibited a peak at 546.3 *m*/*z* for (M+H)^+^, and quantitative analysis, calibrated against the HHT standard, confirmed a content of 43.20 μg/g per mg of extract, as determined from the peak area. Similarly, the compound exhibited a peak at 465.1 *m*/*z* for (M+H)^+^ and 487.1 *m*/*z* for (M+Na)^+^. Quantitative analysis, calibrated against the Q3G standard, confirmed a content of 48.45 μg/g per mg of extract, as determined from the peak area. The standard curves for HHT and Q3G had R^2^ values more than 0.99 (Figure S1A,B).

### 3.3. CHLE Induces Sub-G1 in Liver Cancer Cells

Four cancer cell lines (A549, HCT116, HepG2, and AGS) were treated with CHLE to assess alterations in the cell cycle and the sub-G1-phase. After 24 h of treatment, A549 cells showed a reduction in S-phase (21%) in the CHLE (10 μg/mL) group compared to the DMSO group (Figure S2A). HCT116 cells exhibited decreases in the S-phase (23%) and G2-phase (24%) (Figure S2B). HepG2 cells had a significant increase in the sub-G1-phase and a decrease in the G2-phase (25%) (Figure S2C). AGS cells showed an increase in S-phase (124%) but a decrease in G2-phase (24%) (Figure S2D).

**Figure 2:**
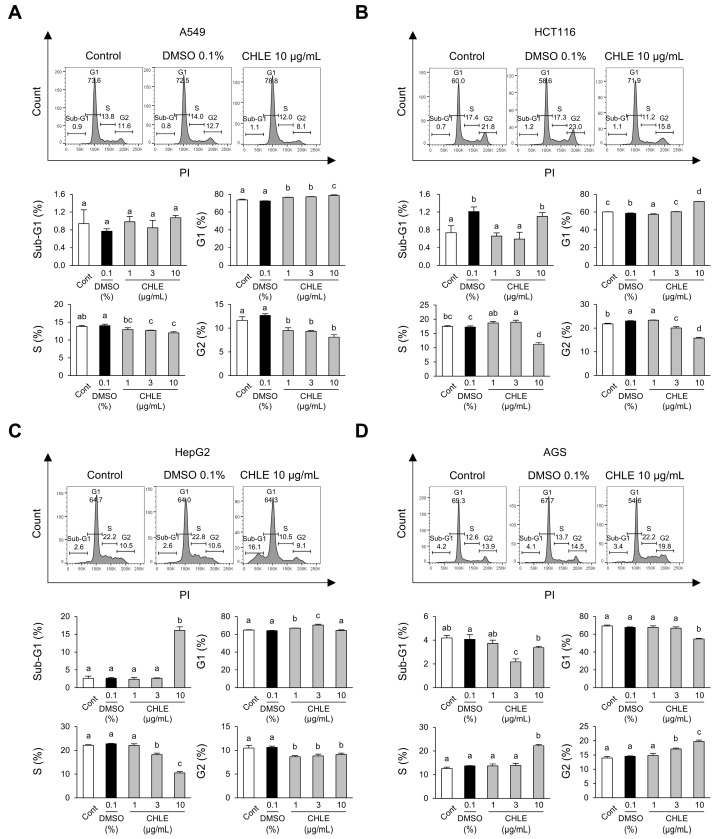
Cell cycle changes at 48 h in four types of cancer cell lines due to CHLE. The cells (5 × 10^4^ cells) were treated with CHLE for 48 h. (**A**) A549 (**B**) HCT116, (**C**) HepG2, and (**D**) AGS cell cycle analysis was performed by flow cytometry. The histograms represent representative experiments, and the values indicate the mean percentages of each phase. Data are presented as the mean ± SD. Different letters (a–d) indicate statistically significant differences (*p* < 0.05).

At 48 h, A549 cells exhibited a reduction in the G2 phase (36%) (Figure 2A), whereas HCT116 cells showed a decrease in both the S-phase (35%) and the G2-phase (31%) (Figure 2B). In HepG2 cells, sub-G1 levels dramatically increased by 616%, while the S-phase decreased significantly by 54% (Figure 2C). AGS cells exhibited increases in the S-phase (162%) and G2-phase (136%) (Figure 2D). The pronounced increase in the sub-G1 indicates DNA fragmentation, a characteristic feature of late apoptosis. Thus, a detailed toxicity evaluation was performed focusing on HepG2 cells, which have a considerably higher sub-G1 population among the four cancer cell types.

### 3.4. CHLE Is Not Cytotoxic in Human Normal Kidney Cell Lines

**Figure 3:**
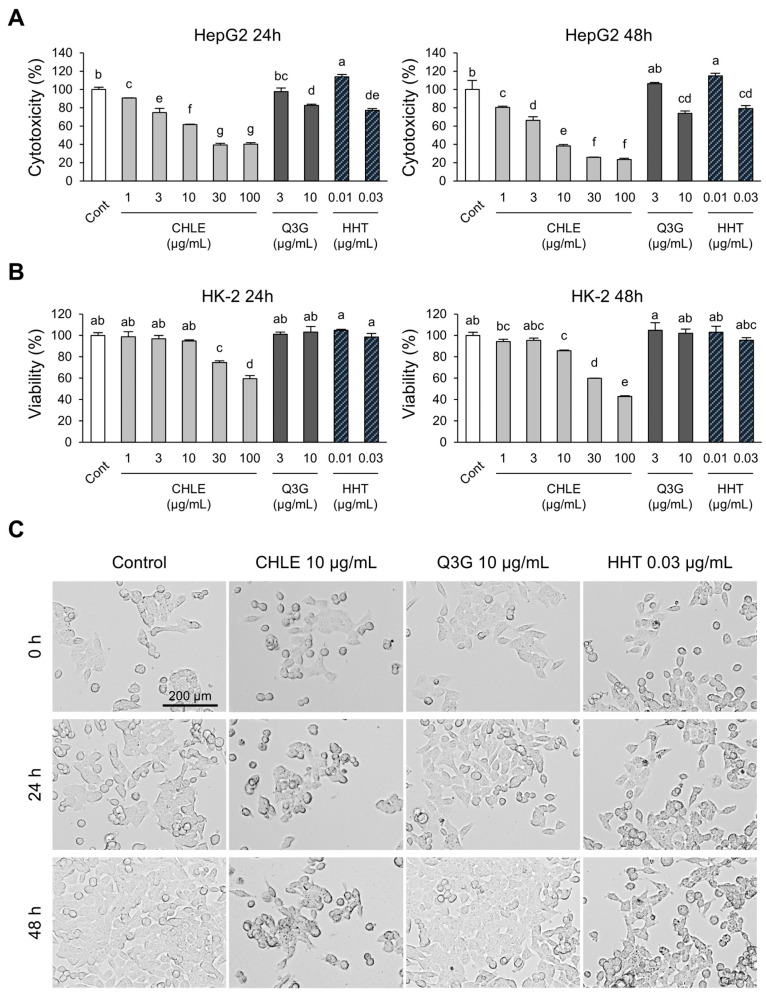
The cytotoxicity effect of CHLE in HepG2 and HK-2 cells. The cells (5 × 10^3^ cells) were treated with CHLE, Q3G, and HHT for 24 h or 48 h. (**A**) HepG2 cells cytotoxicity and (**B**) Viability of HK-2 cells were measured using the Cell Counting Kit-8 (CCK-8). (**C**) HepG2 cells imaged by an EVOS M7000 microscope (×10 objective lens, scale bar: 200 µm). The images presented representative experiments. Data are presented as the mean ± SD. Different letters (a–g) indicate statistically significant differences (*p* < 0.05).

To assess the toxicity of CHLE towards normal cells, the human normal kidney cell line HK-2 was used. In HepG2 cells, the CHLE (10 μg/mL) group showed cytotoxicity at 24 h (38.2%) and 48 h (61.6%), indicating a time-dependent effect (Figure 3A). The main compounds, Q3G and HHT, were confirmed to be significantly cytotoxic at high concentrations (Figure 3A). However, HK-2 cells maintained high viability of over 85% in the CHLE (10 μg/mL) group at both 24 and 48 h, and no apparent cytotoxicity was observed with either Q3G or HHT (Figure 3B). Since CHLE exhibited substantial toxicity in HK-2 cells starting from 30 μg/mL, concentrations of 1, 3, and 10 μg/mL were chosen for subsequent experiments (Figure 3B). Real-time imaging of HepG2 cell proliferation after CHLE treatment showed significantly reduced cell division compared to the control group (Figure 3C, Figure S3). This decrease in proliferation is supported by reduced Cyclin E1 (Figure S4A) and CDK2 mRNA levels (Figure S4B), a consequence of G1 arrest, as shown in Figure 2C. Thus, these findings indicate that CHLE induces both cell cycle regulation and apoptosis.

### 3.5. Apoptosis of HepG2 Cells Induced by CHLE

**Figure 4:**
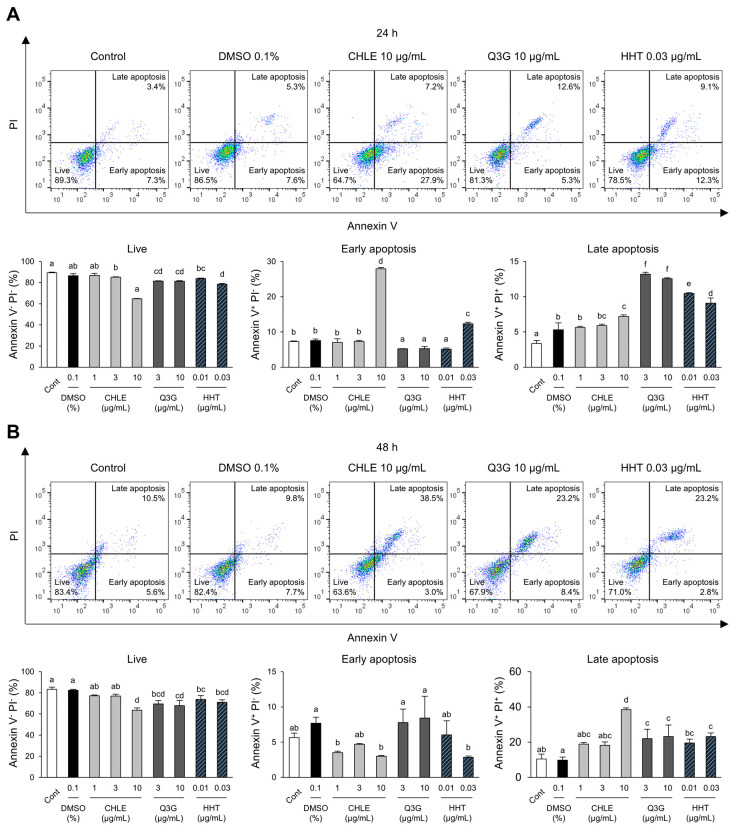
The apoptosis of CHLE in HepG2 cells. The cells (5 × 10^4^ cells) were treated with CHLE, Q3G, and HHT for 24 h and 48 h. (**A**) At 24 h (**B**) and 48 h, the early and late apoptosis were analyzed by flow cytometry. The dot blot presented representative experiments, and the numbers indicate the mean values. Data are presented as the mean ± SD. Different letters (a–f) indicate statistically significant differences (*p* < 0.05).

A significant increase in the sub-G1 stained with propidium iodide (PI) was observed in the cell cycle analysis, rising from 5.2% at 24 h to 16.1% at 48 h. At the same time points, early and late apoptosis were analyzed using Annexin V and PI staining. After 24 h of treatment, early apoptosis increased by 367% in the CHLE (10 μg/mL) group compared with the DMSO group, whereas late apoptosis showed no significant change (Figure 4A). At 48 h, the CHLE (10 μg/mL) group showed a transition from early to late apoptosis (Figure 4B). The major compounds Q3G and HHT also enhanced apoptosis. Overall, CHLE induced cell cycle arrest and apoptosis in liver cancer cell lines.

### 3.6. Activation of the Mitochondrial-Mediated Intrinsic Pathway by CHLE

**Figure 5:**
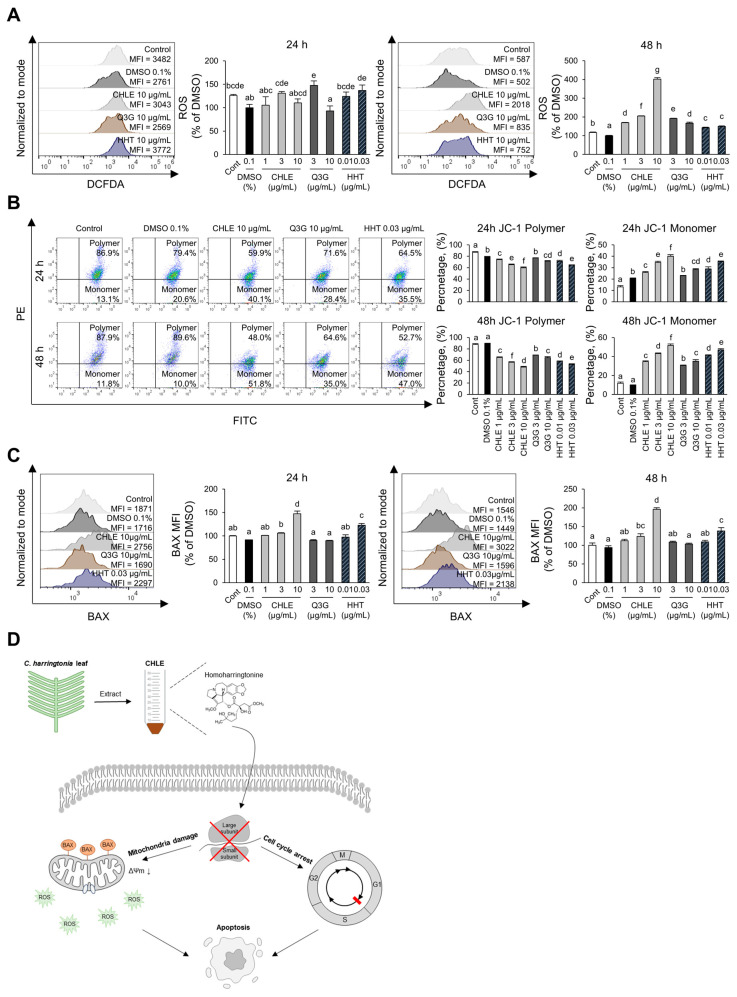
Activation of the intrinsic apoptosis pathway due to CHLE. The HepG2 cells (5 × 10^4^ cells) were treated with CHLE, Q3G, and HHT for 24 h or 48 h. (**A**) Intracellular ROS formation was measured by flow cytometry. (**B**) Mitochondrial membrane potential changes were analyzed by flow cytometry using JC-1 staining. (**C**) Intracellular BAX expression levels were measured by flow cytometry. (**D**) Schematic diagram of CHLE-dinduced apoptosis in HepG2 cells. The histograms and dot blot presented representative experiments, and the numbers indicate the mean values. Data are presented as the mean ± SD. Different letters (a–g) indicate statistically significant differences (*p* < 0.05).

To investigate whether CHLE induces apoptosis through mitochondria-mediated intrinsic pathway in HepG2 cells, key associated factors, ROS formation, MMP changes, and BAX expression level were analyzed. ROS levels, indicative of mitochondrial damage, increased 4.0-fold in the CHLE (10 μg/mL) group compared with the DMSO control after 48 h of treatment (Figure 5A). JC-1 dye exhibits red fluorescence in healthy mitochondria with high membrane potential and green fluorescence in depolarized mitochondria with low membrane potential. CHLE (10 μg/mL) treatment reduced MMP by more than 50% at 48 h, and HHT produced a similar effect (Figure 5B). Mitochondrial damage is mediated due to mitochondrial membrane binding of BAX. BAX expression is upregulated by 1.47-fold at 24 h, and by 1.96-fold at 48 h (Figure 5C). Also, HHT showed a similar effect (Figure 5C). Anti-apoptotic protein BCL-2 expression also increased (Figure S5A). However, the BAX/BCL-2 MFI ratio was higher at 48 h than at 24 h (Figure S5B), indicating that apoptotic signaling was continuing. Comprehensively, HHT contained in CHLE activates cell cycle arrest and the mitochondria-mediated intrinsic pathway by inhibiting protein translation, and ultimately induces apoptosis in HepG2 cells (Figure 5D).

## 4. Discussion

Despite ongoing advances in cancer research, it remains one of the leading causes of death worldwide. Cancer therapy is generally classified into surgical, radiation, immunological, genetic, and chemotherapeutic approaches [39,40]. Among these, anticancer drug treatments are the most commonly used for patients, but they can also cause serious side effects due to their toxicity to normal cells. Therefore, there has been increasing interest in plant-derived extracts, which have long been used for their therapeutic properties and low toxicity [6]. In this study, CHLE was shown to induce cell cycle arrest and apoptosis in human liver cancer cell lines (HepG2).

Apoptosis initiation is categorized into the extrinsic pathway (death receptor) and the intrinsic pathway (mitochondrial). In particular, apoptosis by the intrinsic pathway is considered an effective strategy in cancer therapy [41]. It is activated by sensing external cellular stress through the BH3-only family of proteins [41,42]. The BH3-only family of proteins inactivates anti-apoptotic B-cell lymphoma 2 (BCL-2), promoting the oligomerization of pro-apoptotic BCL-2-associated X protein (BAX)/BCL2 antagonist/killer 1 (BAK), and binds in the mitochondrial outer membrane [43]. This increases mitochondrial outer membrane permeabilization (MOMP), which releases cytochrome c and SMAC/OMI into the cytosol and activates apoptosis [41,42]. Additionally, apoptosis is associated with a decrease in mitochondrial membrane potential (MMP or ΔΨm) and accumulation of reactive oxygen species (ROS) within cells [44,45]. Cells recognizing apoptotic signals translocate phosphatidylserine (PS) to the outer layer of the cell membrane and simultaneously induce internal changes such as DNA fragmentation and chromatin condensation through caspase activation [46,47].

In this study, CHLE (10 μg/mL) significantly increased sub-G1 in liver cancer cells while showing minimal toxicity in normal human kidney cells (HK-2). CHLE increased annexin V-stained apoptotic cells through two mechanisms in liver cancer cells. First, it regulated the cell cycle to induce the G1-phase arrest, as confirmed by real-time imaging that showed reduced cell division. Second, it activated the intrinsic pathway, leading to BAX upregulation, decreased MMP, and increased intracellular ROS levels. Overall, CHLE demonstrated effective anticancer activity through mitochondrial pathway activation and cell cycle regulation. The main compound, Q3G, decreased MMP and induced apoptosis, while HHT induced BAX and apoptosis upregulation, and MMP reduction. Our data showing CHLE-induced mitochondrial depolarization and BAX upregulation align well with these known mechanisms of HHT. Furthermore, the findings suggest a possible synergistic or additive effect between HHT and other compounds present in CHLE. The advantage of a complex plant extract is that multiple bioactive constituents can act on upstream regulators such as antioxidant systems, kinase signaling, and transcription factors, collectively sensitizing tumor cells to apoptosis. This synergy could explain why CHLE demonstrated stronger cytotoxicity than equivalent doses of isolated HHT or Q3G in our assays. Concretely, CHLE appears more effective than its main constituents in inducing apoptosis, likely due to its multiple bioactive components, including anticancer agents such as HHT, harringtonine [48], and ginkgetin [49]. Therefore, CHLE shows potential as a natural and safe candidate for liver cancer treatment and prevention, and the FDA-approved drug HHT may also be further developed as a targeted therapeutic for liver cancer.

## 5. Conclusions

*C. harringtonia* leaf extract was found to selectively inhibit hepatocellular carcinoma (HepG2) cells by inducing G1-phase arrest and activating mitochondrial-dependent apoptosis. The extract increased intracellular ROS levels, disrupted mitochondrial membrane potential, and elevated BAX expression, confirming activation of the intrinsic apoptotic pathway. Its major compound, HHT, contributed to these effects, with evidence suggesting synergistic interactions within the extract. These mechanistic findings provide molecular support for the ethnomedicinal use of *C. harringtonia* in conditions resembling tumor-like disorders. Collectively, *C. harringtonia* leaf extract represents a promising natural source of bioactive compounds for future development in liver cancer prevention and therapy.

## Data Availability

The original contributions presented in this study are included in the article/Supplementary Materials. Further inquiries can be directed to the corresponding author.

## Supplementary Materials

The following supporting information can be downloaded at: https://www.mdpi.com/article/10.3390/cimb47120994/s1.

## Abbreviations

The following abbreviations are used in this manuscript:

*C. harringtonia*	*Cephalotaxus harringtonia*
CHLE	*C. harringtonia* leaf extract
HHT	Homoharringtonine
Q3G	Quercetin 3-β-D-glucoside
ROS	Reactive oxygen species
MMP	Mitochondrial membrane potential
CML	Chronic myeloid leukemia
BAX	BCL-2-associated X protein (BAX)/BCL2 antagonist/killer
PI	Propidium iodide
DCFDA	2′,7′-dichlorodihydrofluorescein diacetate
