# Supplementary material for: Therapeutic Effects of Cephalotaxus harringtonia Leaf Extract on Hepatocellular Carcinoma via Regulation of the Intrinsic Apoptosis Pathway and Cell Cycle
Source: Curr Issues Mol Biol. 2025 Nov 27;47(12):994. doi: 10.3390/cimb47120994 (PMC12731618; doi:10.3390/cimb47120994)
Supplement: Supplementary file 1 [file cimb-47-00994-s001.zip › cimb-3969652-supplementary.pdf]

Supplementary Materials

Therapeutic Effects of *Cephalotaxus harringtonia* Leaf Extract on Hepatocellular Carcinoma via Regulation of the Intrinsic Apoptosis Pathway and Cell Cycle

Dae-Han Park [1,†], Sonny C. Ramos [1,†], Hyun Bo Sim [1], Ju-Bin Lee [1], Ho-Yeol Jang [2], Beom-Gyun Jeong [2], Kyung-Wuk Park [2], Kyung-Yun Kang [2,3,*] and Jong-Jin Kim [1,*]

[1] Department of Biomedical Science, Sunchon National University, 255 Jungang-ro, Suncheon-si, 57922, Republic of Korea

[2] R&D Team, Suncheon Research Center for Bio Health Care, Suncheon-si 57962, Republic of Korea

[3] Future of Natural New Materarials, Room 605, Industry-Academic Cooperation Building, 255, Jungang-ro, Suncheon-si 57922, Republic of Korea

[*]Correspondence: nms-kang@nate.com (K.-Y.K.); kimjj@scnu.ac.kr (J.-J.K.)

[†]These authors contributed equally to this work.

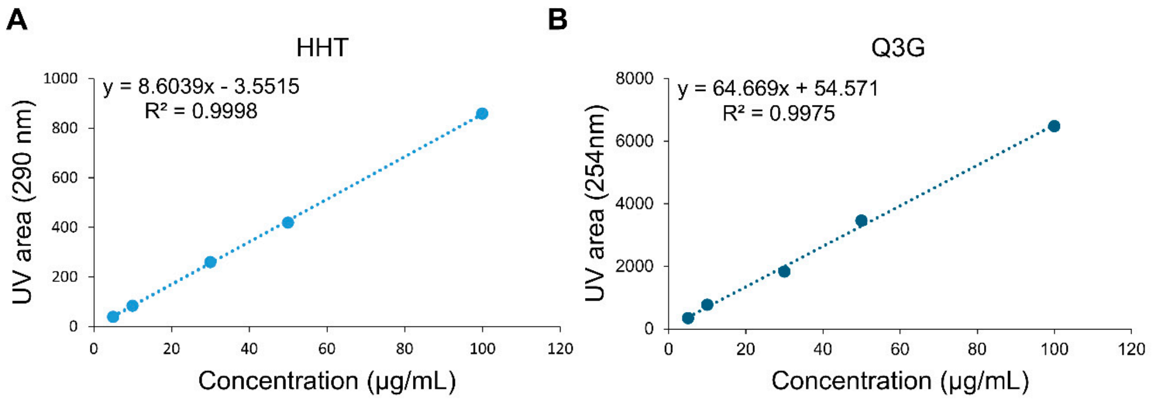

Figure S1. Homoharringtonine and Quercetin 3-β-D-glucoside standard calibration curve. (**A** and **B**) A five-point standard calibration curve was constructed using five different concentrations of the standard material within the range of 5–100 μg/mL.

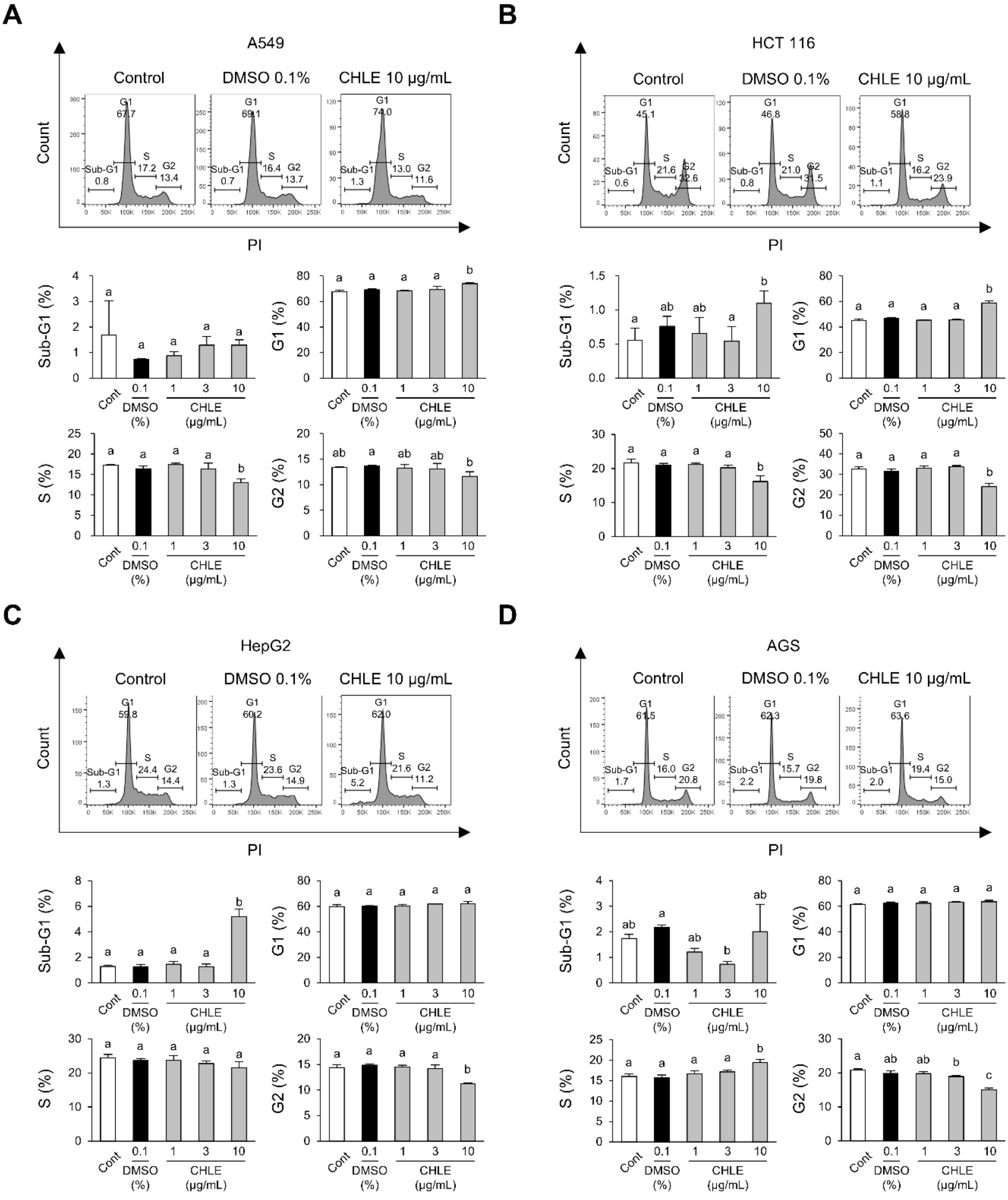

Figure S2. Cell cycle changes at 24 h in four types of cancer cell lines due to CHLE. The cells (5 × 10^4 cells) were treated with CHLE for 24 h. (**A**) A549, (**B**) HCT 116, (**C**) HepG2, and (**D**) AGS cell cycle analysis was performed by flow cytometry. The histograms presented representative experiments, and the numbers indicate the mean values. Data are presented as the mean ± SD. Different letters (a-c) indicate statistically significant differences (*p<0.05*).

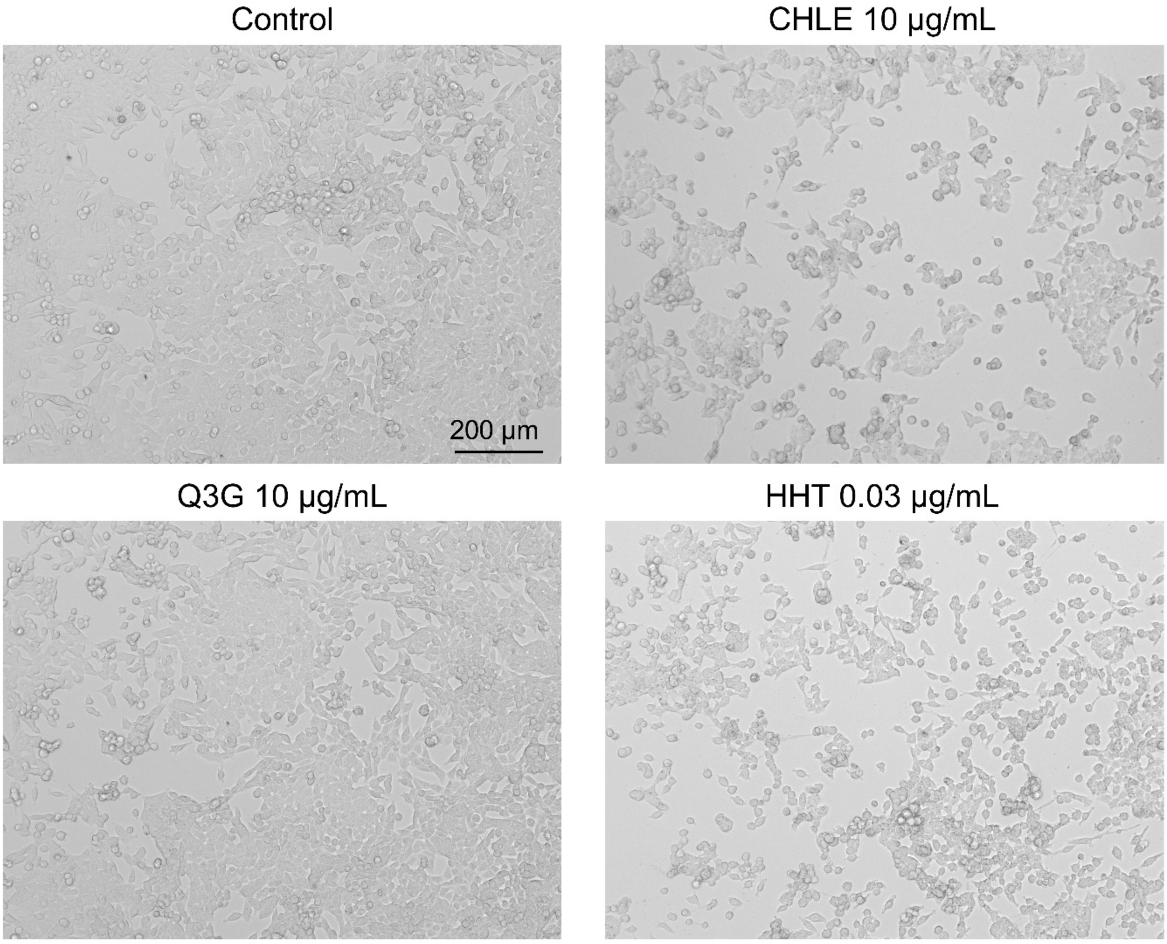

Figure S3. The real-time video of CHLE in HepG2 cells. The HepG2 cells (5 × 10^3 cells) were treated with CHLE, Q3G, and HHT for 48 h. The real-time video was captured using an M7000 microscope (×10 objective lens). The videos represent representative experiments.

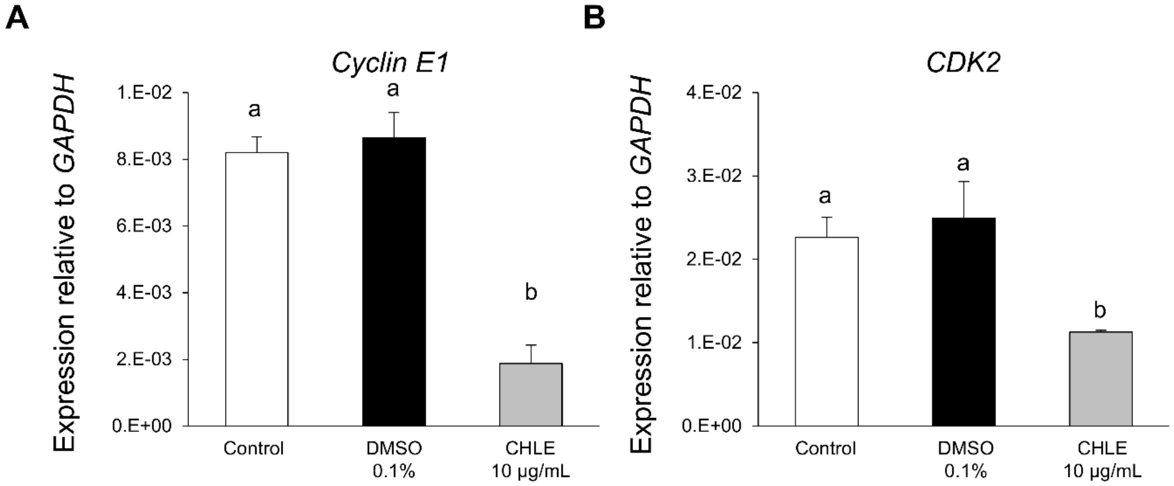

Figure S4. Evaluation of cell cycle regulatory gene expression. The HepG2 cells (5 × 10^5 cells) were treated with CHLE for 24 h. (**A**) Cylin E1 and (**B**) CDK2 mRNA expression level quantified by qRT-PCR. Data are presented as the mean ± SD. Different letters (a-b) indicate statistically significant differences (*p<0.05*).

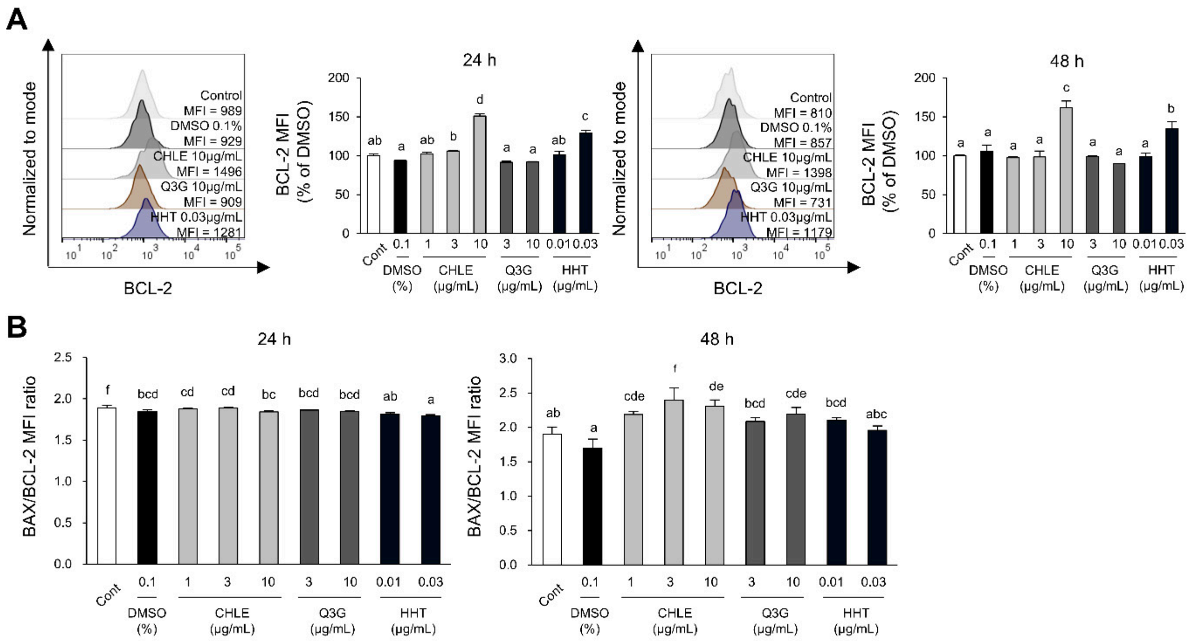

Figure S5. Evaluation of BCL-2 protein expression. The HepG2 cells (5×10^4 cells) were treated with CHLE for 24 h or 48 h. (**A**) Intracellular BCL-2 expression levels were measured by flow cytometry. (**B**) The ratio was calculated by dividing BAX MFI and BCL-2 MFI. The dot blot presented representative experiments, and the numbers indicate the mean values. Data are presented as the mean ± SD. Different letters (a-f) indicate statistically significant differences (*$p<0.05$*).
